# Health among disaster survivors and health professionals after the Haiyan Typhoon: a self-selected Internet-based web survey

**DOI:** 10.1186/s12245-017-0139-6

**Published:** 2017-03-29

**Authors:** Karin Hugelius, Mervyn Gifford, Per Örtenwall, Annsofie Adolfsson

**Affiliations:** 1 School of Health Sciences, Örebro University, Örebro, Sweden; 2 Karlskoga Hospital, Örebro County Council, Karlskoga, Sweden; 3 Sahlgrenska Akademin, Gothenburg University, Gothenburg, Sweden; 4 Prismahuset, Orebro Universitet, 70182 Örebro, Sweden

**Keywords:** Disaster, Natural disaster, Disaster medicine, Disaster response, Mental health, Health professionals

## Abstract

**Background:**

Natural disasters affected millions of people worldwide every year. Evaluation of disaster health and health response interventions is faced with several methodological challenges. This study aimed (1) to describe survivors’ and health professionals’ health, 30 months after a natural disaster using a web-based self-selected Internet sample survey designed and (2) to evaluate the health effects of disaster response interventions, in the present study with a focus on disaster radio.

**Methods:**

A web-based survey was used to conduct a cross-sectional study approximately 30 months after typhoon Haiyan. The GHQ-12, EQ-5D-3L, and EQ-VAS instruments were used in addition to study-specific questions. A self-selected Internet sample was recruited via Facebook.

**Results:**

In total, 443 survivors, from what 73 were health professionals, participated in the study. The Haiyan typhoon caused both physical and mental health problems as well as social consequences for the survivors. Mental health problems were more frequently reported than physical injuries. Health professionals reported worse overall health and a higher frequency of mental health problems compared to other survivors.

**Conclusions:**

There were short-term and long-term physical, psychological, and social consequences for the survivors as a result of the Haiyan typhoon. Mental health problems were more frequently reported and lasted longer than physical problems. Health professionals deployed during the disaster reported worse health, especially concerning mental health problems. The survey used was found useful to describe health after disasters.

## Background

From a historical perspective, natural disasters have always been a threat to human existence. Health effects from natural disasters depend on several factors, among them geographic, economic, and pre-disaster health situations as well as the disaster response [1]. In this study, a biopsychosocial perspective on health [2] was used. The concept has strongly influenced the WHO’s definition of health [3].

In November 2013, typhoon Haiyan (locally called Yolanda) hit parts of the Philippines, affecting over 14 million people. The typhoon caused approximately 7000 deaths and injured approximately 24,000 people, most of them in the area of Tacloban, the provincial capital of Leyte [4]. Physical injuries caused by natural disasters may include traumatic injuries and associated conditions [5]. An increased risk of non-traumatic health problems associated with natural disasters has also been reported [6]. Mental health problems reported include conditions such as stress-related reactions, post-traumatic stress disorder (PTSD), and depression or anxiety. Additionally, disrupted social relations, economic stress, and temporary or permanent displacement are common after natural disasters [7].

The capacity of the local health system to adapt to a disaster situation is essential for post-disaster health [8]. However, little attention has been focused on the health of professionals who are in place during and after disasters [9, 10]. Therefore, health professionals were assessed as a subgroup of survivors in this study.

The knowledge of the health effects of disaster response interventions is limited [11]. Such evaluations entail several methodological challenges, including rapid access to the area and recruitment of participants [12, 13] as well as difficulties to conduct randomized, controlled and ethically approved studies in a disaster area [14]. As part of the general disaster response strategy after the Haiyan typhoon, disaster radio broadcasts were used for the first time as part of the disaster response strategy. The term “disaster radio” refers to a temporary radio station broadcasting disaster-specific information and music in an affected area. Disaster radio was, among other things, used to communicate health information and psychosocial support to the affected community [15] and was experienced by survivors to contribute to recovery [16]. Since this was the first time disaster radio had been used as a specific response intervention, there was a need to explore its possible effects in a health perspective.

This study aimed (1) to describe survivors’ and health professionals’ health 30 months after a natural disaster using a web-based survey designed and (2) to evaluate the health effects of disaster response interventions, in this study with a focus on disaster radio.

## Methods

Data collection was performed approximately 30 months after the Haiyan supertyphoon in the Tacloban area, Leyte province, the Philippines using a cross-sectional web-based survey.

### Sample and recruitment strategy

A self-selected Internet sample was used. For inclusion, the person should be over 18 years old and that they should have experienced typhoon Haiyan. If a potential participator was found not to match the inclusion criteria’s, the survey closed down. No specific exclusion criteria were used.

An invitation to participate in the survey was posted on Facebook. Over 10 days, a short invitation text was posted on five different public Facebook group pages and, to our knowledge, further spread to at least two private Facebook accounts and one closed forum. Facebook users who clicked on the invitation text were provided with the full study information, an opportunity to give their informed consent, and a web link to the survey. The survey was conducted in English, one of the official languages of the Philippines, understood by approximately 97% of the adult population [17]. No monetary or other compensation was offered to the participants. A more detailed description of the recruitment strategy has been reported elsewhere [18].

### Data collection procedure

Data were collected via a web-based survey platform. Using either computer, tablet, or mobile (smart) phone. The data were automatically transferred to Excel format and then imported into the SPSS software package (IBM Corp. Released 2016. IBM SPSS Statistics for Windows, Version 23, Armonk, NY, IBM Corp) for analysis. The survey was anonymous, and no personal data such as name, IP address, e-mail address, or other personal information were requested, saved, or tracked.

### Evaluation instruments

#### GHQ-12

The General Health Questionnaire 12 items version (GHQ-12) is a validated instrument that is commonly used to screen for general mental health among adults [19, 20]. The instrument has been used in post-disaster settings [21] and has been translated into 38 languages, including an English version specifically for use in the Philippines. The instrument consists of six items measuring inability to undertake normal functions and six items on the appearance of new and distressing phenomena. Likert scoring (0-1-2-3) was used in this study [22]. The possible total scores ranged from 0 to 36. A score of 15 or more points was used as a cut-off for affected mental health. More than 19 points was considered to indicate severely affected mental health [23]. Permission to use GHQ-12 was obtained from GL Assessment.

#### EQ-5D-3L

EuroQol Five Dimension Three Level (EQ-5D-3L) is an instrument that is widely used to assess health-related quality of life [24]. It has been used in a wide range of contexts, including disasters [25], and has been officially translated into 172 languages. The EQ-5D-3L comprises five dimensions: mobility, self-care, usual activities, pain/discomfort, and anxiety/depression. In this study, the three-level web version in English for the Philippines was used. EQ-5D-3L also includes a self-reported visual analogue scale (EQ-VAS) that measures overall self-perceived health-related quality of life. It may be converted into a specific index that is used mainly in cost utility analysis. Value sets for a representative sample of the total population are available for several countries. If no country-specific value set is available, it is recommended to use the value sets of the United Kingdom [24]. Permission to use EQ-5D-3L was obtained from the EuroQol Research Foundation.

The informants were also asked 27 study-specific questions covering demographic information (gender, age, education level, profession) and personal experiences related to the disaster event (loss of family member, whether the informant had suffered any physical injuries related to the disaster, whether the informant had suffered any psychosocial/mental health problems related to the disaster, whether or not the informant had listened to disaster radio after the disaster, and whether the informant had been deployed during and/or immediately after the disaster and, if so, as a voluntary worker, health professional or other professional). Additionally, informants were requested to evaluate the survey. The questions could be answered with “yes”, “no”, “do not know/do not remember” or open answer (profession and comments on the survey).

A pilot study was performed before the original study was conducted. Ten disaster survivors from the study area were asked to answer the web survey, using the same technical platform, and to provide feedback on both the content and technical aspects of the survey. No changes except language and grammar corrections were made as a result of the pilot study.

### Statistical analysis

To compare subgroups (health professionals compared to other survivors), Student’s *t* test, chi-square test, and Fisher’s exact test were used as appropriate [26]. Multiple linear regression was used to analyze variables influencing the respondents’ overall health. EQ-VAS was used as the primary outcome value [27]. The factors used for the multiple linear analysis included gender, age, level of education, deployment, physical injuries, mental health problems, loss of family member, use of social support, forced to move, and listened to disaster radio. Residual diagnostics were performed, and the analysis was considered as well fitted. If a participant had missing values in any dimension in GHQ-12 or EG-5D-3L, his or her total score was excluded [22, 28]. The EQ-5D-3L index was calculated in Excel, using the TTO value set for the UK [29]. External statisticians were consulted for the statistical analysis.

## Results

### Characteristics of participants

**Fig. 1:**
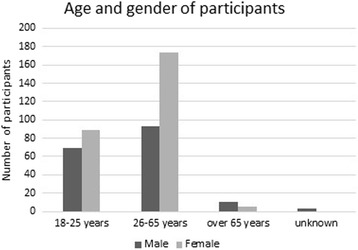
The distribution of age and gender in the whole study sample

In total, 443 participants were included in the study. Of these, 172 (39%) were male and 268 (61%) were female. The majority were adults (*n* = 263, 59%) or young adults between 18 and 25 years old (*n* = 162, 37%), and 18 (4%) of the participants were 66 years or older. The majority, 337 (76%) of the study participants, had been staying in the Tacloban area (an urban area) at the time of the typhoon. Eleven (3%) of the participants had elementary school as their highest completed level of education, 190 (43%) had high school, and 235 (53%) had university studies. Four participants (1%) answered “other level of education”. Of the sample, 73 (17%) were health professionals who had been deployed during the disaster. Additionally, 26 (6%) had been deployed in other professions, and 18 (4%) had been involved in the response as voluntary workers. The distribution of age and gender is shown in Fig. 1.

### Internal dropouts

All 443 participants filled in the GHQ-12 questions, while 3 participants failed to answer all questions in EQ-5D-3L and 14 did not answer the EQ-VAS. For the study-specific questions, the missing number of answers varied from 0 (4 questions) to 100 (the open-answer question about profession), with a median of five missing answers. The questions about psychosocial problems/mental health (38 missing answers), use of disaster radio (29 missing answers), and use of social support (23 missing answers) had the highest number of internal dropouts, except for the open-answer question about profession.

### Physical health after the disaster

Of all survivors, 72 (16%) stated that they had suffered physical injuries related to the typhoon at any time from the typhoon to the time of data collection (30 months). After 30 months, 20 (5%), stated that they still suffered from their physical injuries. None of the participants reported severe problems with moving around, taking care of themselves, or conducting their usual activities, but some reported problems with pain/discomfort (see Table 1).

**Table 1:** EQ-5D-3L health 30 months after typhoon Haiyan

EQ-5D-3L Dimensions	Scoring	18–25 years *n*	26–65 years *n*	≥66 years *n*	Total %
Mobility	No problems	159	248	10	95%
	Some problems	2	14	7	5%
	Severe problems	0	0	0	0%
Self-care	No problems	161	262	15	99.5%
	Some problems	0	0	2	0.5%
	Severe problems	0	0	0	0%
Usual activities	No problems	154	238	11	91.5%
	Some problems	7	23	6	8.5%
	Severe problems	0	0	0	0%
Pain/discomfort	No problems	149	223	10	87%
	Some problems	12	36	7	12.5%
	Severe problems	0	2	0	0.5%
Anxiety/ depression	No problems	141	198	16	80%
	Some problems	20	63	1	19%
	Severe problems	0	1	0	0.03%
Total *n* = 440^a^		161	262	17	

^a^missing values for 3 participants in the study sample

### Mental health after the disaster

In total, 185 (42%) of the participants stated that they had, at any time, suffered mental health problems related to the typhoon, and 52 (12%) reported persistent mental health problems after 30 months. After 30 months, 21% (*n* = 91) showed affected psychological health (GHQ-12 score of 15 or above), and 6% (*n* = 25) were assessed as having severe mental health problems (GHQ-12 score of 20 or above) (see Table 2).

**Table 2:** Overview of reported health, in total sample, survivors not deployed and health professionals deployed

	Survivors total sample *n* (%)	Survivors not deployed at all or as not as health professionals *n* (%)	Health professionals deployed during the disaster *n* (%)	*p* value survivors not deployed vs. health professionals deployed
Number^a^	*n* = 443	*n* = 365	*n* = 73	
Gender				
Male	172 (39)	151 (41)	20 (27)	0.024
Female	286 (61)	213 (59)	53 (73)	
Age***				
18–25	162 (16)	152 (42)	6 (8)	<0.000
26–65	263 (59)	196 (54)	66 (90)	
66+	17 (4)	16 (4)	1 (1)	
Loss of family members	171 (39)	152 (42)	18 (25)	0.007
Forced to move	251 (57)	223 (61)	27 (37)	<0.000
Listened to radio	296 (67)	276 (79)	19 (29)	<0.000
Used social support	272 (61)	237 (68)	35 (50)	0.004
Physical injuries at any time	71 (16)	66 (18)	6 (8)	0.040
Persistent physical injuries***	20 (5)	20 (7)	0 (0)	0.053
Psychological problems at any time	185 (42)	126 (38)	59 (83)	<0.000
Persistent psychological problems	52 (12)	32 (11)	20 (32)	<0.000
Frequency of affected mental health				
GHQ ≥15	91 (21)	57 (16)	34 (47)	<0.000
GHQ ≥20	25 (6)	18 (5)	7 (10)	0.117
EQ-VAS score^**^				
Mean	70.0	71.0	64.0	<0.001
SD	18.2	18.3	16.3	
Median	73.0	75.0	65.0	
25th	58.0	59.0	50.5	
75th	85.0	85.0	79.5	
GHQ-12 score^**^				
Mean	10.7	10.2	13.6	<0.000
SD	4.8	4.6	4.5	
Median	10.0	9.0	14.0	
25th	8.0	7.0	10.0	
75th	14.0	13.0	16.5	

^a^Five participants could not be categorized due to missing data

*p calculated with chi-square test

**p calculated with *t* test

***p calculated with Fisher’s exact test

### Social consequences

Loss of a loved one was experienced by 172 (39%) of the participants, and 251 (57%) had been forced to move from their homes. At the time of the data collection (30 months), 69 (16%) still lived somewhere other than their usual home. Of the study sample, 272 (61%) had been using social support, described as support from family, friends, neighbors, or colleagues. Following the typhoon, 296 (67%) stated that they had listened to disaster radio.

### Quality of life after the disaster

The overall health, measured with EQ-VAS, was 70. No significant differences in EQ-VAS scoring could be seen between the male and female genders (*p* = 0.574; EQ-VAS male mean 71.53, SD 17.22 and EQ-VAS female mean 69.51, SD 1.7) or between the age of 18–25 and the age of 26–65 (*p* = 0.082; EQ-VAS 18–25 years mean 72.14, SD 17.55 and 26–65 years mean 68.94, SD 18.28), but participants aged 66 and over had significantly lower EQ-VAS scores (*p* = 0.001, 66+ mean 62.53, SD19.9) compared to younger survivors. The EQ-5D-3L index for the whole sample was 0.928 (SD 0.15, median 1.000, 25th 0.848, 75th 1000).

### Factors associated with health outcomes 30 months after the disaster

Multiple linear regression was used to analyze factors influencing health 30 months after the disaster for the whole study sample. The overall health (EQ-VAS) of the whole study sample was positively influenced by being deployed as a voluntary worker in comparison to not being deployed as well as if the person had been listening to disaster radio. It was negatively influenced by the loss of a family member, reported mental health problems or physical injuries, or a lower level of education level (high school) in comparison to university (see Table 3). The regression model was significant (*p* < 0.000), explaining 31.4% of the model.

**Table 3:** Factors influencing EQ-VAS 30 months after the disaster

	Unstandardized coefficients		
	B	95% CI for B (lower bound; upper bound)	Sig.
Gender	1.260	(−2.48; 5.00)	.507
Age			
18–25 years	7.331	(−1.33; 16.00)	.097
26–65 years	6.334	(−1.95;14.60)	.134
Highest level of education			
Elementary school	.666	(−11.35;12.69)	.913
High school	−4.011	(−7.90; 1.13)	^a^.043
Deployed during typhoon			
As health professional	−3.019	(−8.66; 2.62)	.293
As other professional	3.508	(−3.72; 10.739)	.341
As voluntary worker	9.946	(2.33;17.56)	^a^.011
Physical injuries	−6.292	(−11.08; −1.51)	^a^.010
Psychological problems	−8.698	(−12.32; −5.08)	^a^.000
Use of social support	.690	(−3.04; 4.42)	.716
Loss of family member	−9.932	(−13.69; −6.18)	^a^.000
Forced to move			
Still living elsewhere	−5.486	(−11.01; .04)	.520
Returned home	−2.303	(−5.98; 1.37)	.218
Listened to disaster radio	8.387	(4.36; 12.42)	^a^.000

Multiple linear regression, *R* = 31,4

^a^significant value

### Health among health professionals

In the sample, 73 (17%) of the participants stated that they had been deployed as health professionals during and immediately after the disaster event. When comparing their health to survivors not deployed, a significantly lower overall health (EQ-VAS) was reported. Also, mental health problems at any time as well as persistent problems were significantly more common among health professionals as compared to other survivors. No significant difference could be observed for physical injuries. There was no significant difference regarding gender between the two groups. However, the group of health professionals had less young adults than the group of other survivors (see Table 2).

GHQ-12 score was also lower among health professionals in comparison to other survivors. More health professionals were reporting affected mental health (GHQ >14) while no such difference could be seen for those severely affected. (GHQ >19) (see Table 2).

## Discussion

This study showed that the Haiyan typhoon had physical, mental, and social health effects in both a short- and long-term perspective. Health professionals, as a subgroup of survivors, reported worse health and a higher frequency of affected mental health.

Approximately 16% of the survivors reported physical injuries related to the typhoon. The most of these seemed to have recovered, and none reported severe problems in mobility, self-care, or performance of their usual activities 30 months after the disaster. However, it should be noted that persons dying from their injuries during the 30-month period or survivors with severe sequele of their injuries (i.e. traumatic brain injuries) would not have been able to participate in a study like this. Also, the survey did not specifically address non-traumatic physical health problems. The frequency of physical health problems could have been underestimated.

In this study, about 1/5 of the study population had affected mental health 30 months after the disaster event, as measured with GHQ 12. Also, in the EQ-5D-3L psychological dimension, 19% reported problems, but only 12% answered “yes” to the direct question “*Have you ever suffered from psychosocial/mental health problems related to the typhoon?*” This discrepancy between the outcome of the instruments and self-reported frequency can be influenced by many factors. There are many specific disaster mental health outcomes, such as PTSD or depression, that can be measured in disaster mental health studies. However, mental health is a state of well-being, not merely the absence of illness, and therefore, a broader perspective than specific diagnosis was used in this study. The meaning of mental health, or health in general, might include aspects not covered by instruments but still of importance to the individual perception of health [30, 31].

Some studies have indicated that convenience samples relating to psychological well-being after traumatic events are more likely to overestimate pathology as compared to population-based samples [7]. Others have suggested the opposite [32]. Several factors, such as a previous history of mental illness, level of perceived threat to life, physical injuries, and social support [33, 34], have been found to influence mental health after traumatic events. In this study, some of these factors were not assessed, and therefore, the results must be interpreted with caution. The loss of a loved one was the factor that most negatively influenced general health. Although the exact underlying mechanism of social relations and support in disaster situations is still not fully known, it has been observed that such aspects are of importance for health [21, 35].

The EQ-VAS among the total study sample was 70. No comparable data for survivors of natural disasters or the Philippines have been found. Without such data, this result is hard to evaluate [24, 27]. In the analysis of factors influencing EQ-VAS, physical injuries, psychological problems, and social consequences had a significant impact. This supports the idea that assessing and promoting health after disasters should both include physical, psychological and social dimensions.

The functioning of the health care system is vital in reducing negative health effects on the population in most disasters. The burden on the health care system will last for a long time after a disaster event [36]. Therefore, the well-being of health professionals is important both in a short- and long-term perspective. Our study showed that health professionals reported worse overall health and a higher frequency of mental health problems. Professional rescue personnel have been shown to be at greater risk for PTSD than the general population after disasters [37]. This survey does not allow any detailed description of the mental health problems the population sample suffered from. However, the finding indicates a need for further studies on health among health professionals deployed during a disaster, as also suggested also by Chan et al. [38].

Evaluation of health effects from specific disaster repose interventions is a challenge as many factors can influence and interfere [13]. Still, there is a strong need of more evidence on the use and effects from disaster response interventions. In this study, there seemed to be positive health effects from listening to disaster radio, but since this was the first time disaster radio had been used, and the study sample was based on self-selection, further studies are needed to explore possible correlations. Though, the methodology used to evaluate specific disaster response interventions need to be further developed, and this study could contribute to such evaluations in the future.

### Methodological considerations and limitations

The nature of disasters entails several methodological challenges, including sampling strategies, randomization and data collection procedures. The use of a self-selected Internet sample survey has advantages in disaster settings, such as reducing the need to physically locate the researcher in the stricken area. It allows follow-ups regardless of physical location of the participant and is able to reach a large number of participants in complex environments and contexts. Additionally, the use of the Internet to conduct surveys on mental health has been shown to offer the participants a more flexible and anonymous way to participate in research studies [39].

The method also has several disadvantages. Selection biases, such as previous health history, level of exposure, or willingness to report health problems, are difficult to control using web-based recruitment strategies [14]. The lack of baseline data is another commonly described issue when describing health impacts and evaluate disaster response interventions [13, 40]. To summarize, generalization from a self-selected Internet sample to the general population and from one disaster to another can be problematic [13]. However, conducting traditional RTC studies or randomized sampling procedures in a post-disaster context is close to impossible [1, 12, 41]. In order to find practical strategies to conduct disaster health research studies, the development and use of alternative methods are important [13]. The present study added experience in this field, but further use and evaluation of web-based strategies are needed.

So far, there is no agreed-upon consensus about timing for disaster health research [12, 40]. The time for data collection in studies on health and recovery after disasters varies from one month to over 15 years [21]. The time elapsed from a disaster event to the time for data collection means that possible confounders and factors that could have influenced the results were not easily detected or accounted for [42]. Although critical situations such as experiencing a disaster are not forgotten easily [43], recall biases must therefore be considered. The time for data collection, 30 months was found suitable to describe long-term effects and could therefore be considered for future studies.

Both the EQ-5D-3 L and the GHQ-12 have been officially translated into many languages. When a disaster may strike an any geographic area, this can be an advantage. The instrument with the highest internal dropout was EQ-VAS (3% dropout rate). The layout of the VAS scale in the web format, demanding that the participant move a marker on a line instead of using check boxes, could have influenced the response rate. The line and size of the marker depended on the screen size used. How that affected the answers or the answering rate cannot be judged from this study. Still, the dropout level was low, and we consider EQ-VAS to be a useful tool to describe overall health. When there was no specific value set for calculating the EQ-5D-3L index for the Philippines, the data set for United Kingdom was used. However, the use of weights in any kind of health studies will never be absolutely neutral [27]. Still, including the EQ-5D-3L index in a survey such as this might enable possibilities for future health economic analysis of disaster response interventions.

Sample sizes in disaster mental health studies vary from eleven to over 5000 participants, with a median of 150 study participants [32]. With this taken into consideration, this study included a comparatively large number of survivors. Despite several limitations regarding the possibilities to generalize and estimate correlations, the findings can contribute to an increased understanding of post-disaster health [39] as well as studies of potential health effects from disaster response interventions in the future [44].

## Conclusions and implications

There were short-term and long-term physical, psychological, and social consequences for the survivors as a result of the Haiyan typhoon. Mental health problems were more frequently reported and lasted longer than physical problems. This finding emphasizes the importance of addressing physical, psychological and social aspects of health when assessing disaster health and interventions to promote health.

Health professionals deployed during the disaster reported worse health than other survivors, especially concerning mental health problems. This indicates a need for further studies of this specific subgroup of survivors who play an important role in the disaster response system. The survey used was found useful to describe health after disasters in a long-term perspective.

## Abbreviations

EQ-5D-3 L: EuroQol Five Dimension Three Level
EQ-VAS: EuroQol Visual Analogue Scale
GHQ-12: General Health Questionnaire 12 items version
PTSD: Post traumatic stress disorder
